# Surgical treatment of rectovaginal fistula—predictors of outcome and effects on quality of life

**DOI:** 10.1007/s00384-022-04206-7

**Published:** 2022-07-02

**Authors:** Erik V. Söderqvist, Peter H. Cashin, Wilhelm Graf

**Affiliations:** Department of Surgical Sciences, Uppsala University, 75185 Uppsala, Sweden

**Keywords:** Rectovaginal fistula, Colorectal surgery, Colorectal diseases, Vaginal diseases, Quality of life

## Abstract

**Purpose:**

To determine the results after rectovaginal fistula (RVF) repair and find predictors of outcome. Primary objective was fistula healing. Secondary outcomes were morbidity and patient health-related quality of life (HRQoL).

**Method:**

An observational study of 55 women who underwent RVF repair including both local procedures and tissue transposition 2003–2018 was performed. Baseline patient and fistula characteristics were registered, combined with a prospective HRQoL follow-up and a general questionnaire describing fistula symptoms.

**Results:**

Healing rate after index surgery was 25.5% (*n* = 14) but the final healing rate was 67.3% (*n* = 37). Comparing the etiologies, traumatic fistulas (iatrogenic and obstetric) had the highest healing rates after index surgery (*n* = 11, 45.9%) and after repeated operations at final follow-up (*n* = 22, 91.7%) compared with fistulas of inflammatory fistulas (Crohn’s disease, cryptoglandular infection, and anastomotic leakage) that had inferior healing rates after both index surgery (*n* = 7, 7.1%) and at final follow-up (*n* = 13, 46.4%). Fistulas of the category others (radiation damage and unknown etiology) included a small amount of patients with intermediate results at both index surgery (*n* = 1, 33.3%) and healing rate at last follow-up (*n* = 2, 66.7%). The differences were statistically significant for both index surgery (*p* = 0.004) and at final follow-up (*p* = 0.001). Unhealed patients scored lower than both healed patients and the normal population in 6/8 Rand-36 domains, but the differences were not statistically significant.

**Conclusions:**

Most traumatic rectovaginal fistulas closed after repeated surgery whereas inflammatory fistulas had a poor prognosis. Low healing rates after local repairs suggest that tissue transfer might be indicated more early in the treatment process. Unhealed fistulas were associated with reduced quality of life.

Trial registration Clinicaltrials.gov No. NCT05006586.

## Introduction

A rectovaginal fistula (RVF) is an abnormal tract connecting the anorectum and vagina which causes major morbidity and psychosocial dysfunction [1].

Obstetrical trauma, infection, and Crohn’s disease are major etiologies [1–6]. Iatrogenic causes are not as common and include surgical trauma, radiation damage, or fistulation related to a rectal anastomosis. Crohn’s related RVFs have an inferior prognosis compared to fistulas of traumatic and infectious origin [2, 6–8].

The results of surgical repair vary and, depending on the method used, healing rates range from 20 to 100% [9]. At times, a protective stoma is added, either before or at the time of repair. This is done to reduce local inflammation and to protect the RVF repair. The added benefit of a stoma has not yet been determined [1, 2, 10, 11].

The aim of this study was to analyze the results of RVF repair and identify factors that influence the healing rate. The effect of adding a protective stoma was likewise assessed. We also studied the effect of fistula healing on the patient’s quality of life.

## Materials and methods

We studied patients treated for RVF between January 2003 and December 2018, at the Department of surgery, Akademiska University hospital, Uppsala, Sweden. The following inclusion criteria were used: verified recto-, ano-, or pouch vaginal fistula and attempt at surgical closure. The cohort was identified using the surgical codes for RVF, but medical records with codes for perianal fistulation were also reviewed to identify potential RVF.

Median time between index surgery and first follow-up was 2.8 months (*IQR* 1.7–4.9) and between index surgery and last follow-up 30.2 months (*IQR* 12.5–51.7). Clinical information was registered from the medical records, including age, body mass index (BMI), smoking habits, current medication including steroids and/or immunosuppressants, and inflammatory bowel disease (IBD) diagnosis at the time of inclusion. Fistula characteristics included etiology, symptoms, fistula size, location, the number of prior surgical repairs, diverting stoma, and draining seton. However, no details of prior surgical repairs from referring hospitals could be extracted because of limited access to referring hospitals files. The fistulas were classified in two main groups according to origin, i.e., inflammatory origin (including IBD, infection, and anastomotic-related fistulation) or traumatic (obstetric or iatrogenic); the remainder were assigned to others (radiation and unknown origin). Iatrogenic fistulation was RVF caused by prior surgical trauma. Index surgery was defined as the first attempt at fistula repair performed at our clinic. The surgical method used as index surgery was noted and assigned to a group corresponding to the approach used, transrectal, transperineal, and others. Complications within the first 30 days postoperatively were recorded and classified using the modified Clavien-Dindo classification system [12].

The study was approved by the Ethics Committee of Uppsala County (Dnr 2020–01,307) and registered at clinical trials.gov (NCT05006586).

### Surgical methods

**Fig. 1:**
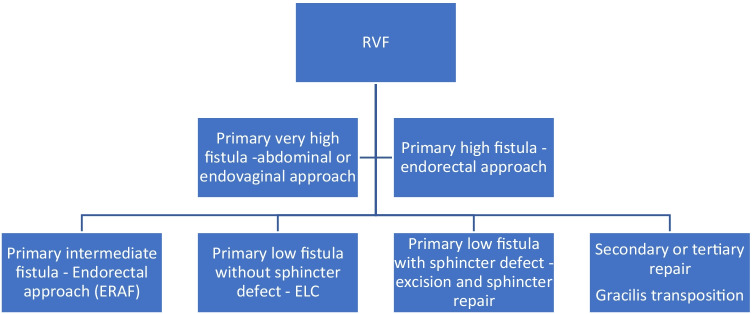
A flow chart illustrating our approach to RVF. In intermediate to high fistulation, an endorectal approach is usually preferred over a transperineal approach. Intermediate fistulation provides the option of several possible methods, the most common being ERAF. Gracilis transposition is most commonly reserved for patients with recurrent fistulation

The fistula tract was examined preoperatively using a probe. In addition, palpation, rectoscopy, and endoluminal ultrasound were routinely used in the preoperative evaluation, occasionally in combination with MRI. Clinical assessment combined with ultrasound was used to classify the fistula as high, intermediate, or low corresponding to above, through the main-, or the lower part of the external sphincter. Very high fistulation (fistula opening located at or near the vaginal vault) was not the main scope of this article. When classifying clinical outcome, the surgeon’s assessment was decisive. Without signs and symptoms of persistent or recurrent RVF at follow-up, the treatment was considered successful. All asymptomatic patients without signs of a fistula at the last follow-up were registered as finally healed. A simplified approach to the process of choosing surgical method is provided in Fig. 1. Transrectal approaches included endorectal advancement flaps (ERAF) [13] and transrectal excision and layered closure (ELC). Transperineal procedures included transperineal ELC, modified ligation of intersphincteric fistula tract (LIFT), overlapping sphincteroplasty, and gracilis transpositions. Other procedures included transvaginal advancement flaps and abdominal procedures (Table 1). All patients received preoperative antibiotic prophylaxis, but bowel preparation was individualized and usually consisted of a small enema. The use of a diverting stoma or draining seton was used at the surgeon’s discretion.

**Table 1:** Surgical methods used at index surgery (*n* = 55)

Method	*n*	(%)
ERAF^a^	24	(43.6)
Rectal ELC^b^	6	(10.9)
Transperineal ELC	9	(16.4)
Sphincteroplasty	5	(9.1)
LIFT^c^	2	(3.6)
Gracilis interposition	1	(1.8)
Others	8	(14.5)

^a^Endorectal advancement flap

^b^Excision and layered closure

^c^Ligation of intersphincteric fistula tract

### Questionnaire

Patients who consented to participate were sent two questionnaires by mail, the RAND-36, which measures the general health-related quality of life (HRQoL). In addition, a short, standardized form was completed with five questions regarding patient satisfaction with the repair performed, any persisting symptoms, whether any subsequent repairs had been performed, and if the patient perceived that their fistula was healed. Lastly, the patient was given an option to provide any additional comments. For the HRQoL follow-up, Swedish normative data were used for comparison [14].

### Statistics

Variables were tested for normality using the Shapiro–Wilk test. Data with normal distribution were presented using mean and ( ±) standard deviation. Otherwise, the median and interquartile range (*IQR*) was used. Categorical data were analyzed using the Fisher-Freeman-Halton exact test of independence. Non-parametric numerical data were analyzed using the Mann–Whitney *U*-test. Physical health component score (PCS) and mental health component score (MCS) were calculated using each RAND-36 domains respective physical factor scoring coefficient. For all analyses, a *P*-value of < 0.05 was considered statistically significant. All collected data were recorded in a database and later processed with the Statistical Package for the Social Sciences (SPSS, Chicago, IL).

## Results

### Patient characteristics

Of 254 patients with surgical codes for perianal or rectovaginal fistulas, 59 patients with RVF were identified. Three were excluded because no attempt of surgical closure was performed, and one patient was excluded since the fistula was a result of a male to female transition. In total, 55 patients were included in this study with a median follow-up time of 30.2 months (*IQR* 12.5–51.7). The majority (*n* = 41) were referred from other hospitals. Twenty-four (43.6%) had undergone at least one previous attempt of fistula closure and five (9.0%) had had three or more previous repairs.

The most common origin was obstetric trauma (*n* = 18, 32.7%) followed by cryptoglandular infection (*n* = 17, 30.9%). Low fistulas were the largest group (*n* = 27, 49.1%), three were high (5.5%), and 17 of intermediate height (30.9%), whereas eight fistulas (14.5%) could not be categorized concerning height.

Most women presented with symptoms of abnormal vaginal discharge (*n* = 34) and/or stool per vagina (*n* = 33). Twenty-four women complained of vaginal flatulence. Eighteen experienced pelvic pain. Nine patients reported recurrent urinary tract infections. The median age was 40 years (*IQR* 33–49) with a mean BMI of 27 kg/m2 (± 5.5). Thirteen (23.6%) patients had a diagnosis of IBD. Of these, 9 (16.3%) patients had Crohn’s disease and 4 (7.3%) had ulcerative colitis.

### Clinical results and predictive factors

The final overall fistula healing rate was 67.3% (*n* = 37), with a healing rate of 25.5% (*n* = 14) after index surgery. A higher ASA class or treatment with an immunomodulant was associated with a lower final healing rate (Table 2). A high fistula (above the external sphincter) had an overall poor prognosis with none healed (*P* = 0.047). Fistulas of the inflammatory category, namely, IBD related, infectious, and anastomotic fistulas, were related to a worse prognosis compared with fistulas of iatrogenic or obstetric origin. i.e., traumatic fistulas (Table 3). This difference was observed for final healing rate (*P* = 0.003) as well as after index surgery (*P* = 0.02). Comparing the different etiologies on a group level, traumatic fistulas had the best results after index (*n* = 11, 45.9%) followed by others (*n* = 1, 33.3%) and finally inflammatory fistulas (*n* = 7, 7.1%). Healing rates on final follow-up showed a similar pattern with traumatic fistulas showing superior results (*n* = 22, 91.7%) followed by fistulas in the others category (*n* = 2, 66.7%) and inflammatory fistulas having the worst results (*n* = 13, 46.4%) The differences were significant for both index (*P* = 0.004) and final follow-up (*P* = 0.001).

**Table 2:** Patient characteristics in relation to fistulas healed *n* (%), after index surgery and at last follow-up

Variable	Total cohort	Healed after index surgery		*P*-value	Healed at final follow-up		*P*-value
Age	*n*	*n* (%)		1.00	*n* (%)		0.39
≤ 40	30	8	(26.7)		22	(73.3)	
> 40	25	6	(24.0)		15	(60.0)	
BMI				0.45			1.00
< 18.5	2	0	(0.0)		1	(50.0)	
18.5–25	20	7	(35.0)		14	(70.0)	
> 25	33	7	(21.2)		22	(66.7)	
IBD				0.35			0.15
No	42	13	(31.0)		31	(73.8)	
Crohn’s	9	1	(11.1)		4	(44.4)	
UC	4	0	(0.0)		2	(50.0)	
Immunomodulants (missing = 16)				0.54			0.03*
None	30	8	(26.7)		23	(76.7)	
Steroids	3	1	(33.3)		2	(66.7)	
Immunosuppressants	5	0	(0.0)		1	(20.0)	
Combined	1	0	(0.0)		0	(0.0)	
Current smoker				0.27			0.59
No	51	12	(23.5)		35	(68.6)	
Yes	4	2	(50.0)		2	(50.0)	
ASA class (missing = 1)				1.00			0.03*
1	28	8	(28.6)		22	(78.6)	
2	24	6	(25.0)		14	(58.3)	
3	2	0	(0.0)		0	(0.0)	
Parity (vaginal) (missing = 17)				0.38			0.39
0	3	0	(0.0)		2	(66.7)	
1	17	7	(41.2)		15	(88.2)	
2	13	3	(23.1)		9	(69.2)	
3	3	2	(66.7)		3	(100)	
4	2	1	(50.0)		1	(50.0)	
Total	55	14	(25.5)		37	(67.3)

**Table 3:** Fistula and surgical characteristics in relation to fistulas healed *n* (%), after index surgery and after last follow-up

Variable	Total cohort	Healed after index surgery		*P*-value	Healed at final follow-up		*P*-value
Etiology							
Traumatic origin							
Obstetric	18	7	(38.9)	0.02*	17	(94.4)	0.003*
Iatrogenic	6	4	(66.7)		5	(83.3)	
Inflammatory origin							
Infection	17	1	(5.9)		9	(52.9)	
Crohn’s	7	1	(14.3)		3	(42.9)	
Anastomotic	4	0	(0.0)		1	(25.0)	
Others							
Unknown	2	1	(50.0)		2	(100.0)	
Radiation	1	0	(0.0)		0	(0.0)	
Approach							
Transrectal	31	9	(29.0)	0.440	22	(71.0)	0.024*
Transperineal	18	5	(27.8)		14	(77.8)	
Other	6	0	(0.0)		1	(16.7)	
Total	55	14	(25.5)		37	(67.3)	

ERAF was the most common index procedure (Table 1). Only one patient with fistulation after radiotherapy underwent an abdominal procedure because of a fistula close to the vaginal top using peritoneal flaps covering the fistula opening. The remainder of the “others” group consisted of transvaginal procedures closing fistulas in the upper vagina. There was no significant difference in fistula closure after index surgery when looking at the surgical approach. However, at final follow-up, there was a significant difference in final healing rate between patients with different surgical approaches at index (Table 3).

**Fig. 2:**
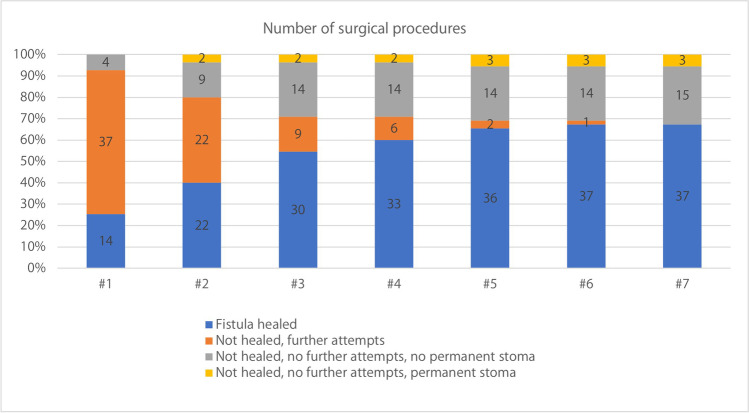
The cumulative percentage (y axis) of healed fistulas correlated to the number of attempts at fistula closure (x axis). Figures within colored fields indicate the number of patients

No significant correlation was found between the number of prior surgical attempts at referring institutions and healing after index surgery. The cumulative effect of additional attempts at our institution and fistula closure is presented in Fig. 2. Looking at the methods used for each individual treatment attempt (*n* = 122), local repair was the most common method and had similar results as ELC. Gracilis transposition was more frequently used in recurrent fistulation than at index surgery and had a superior healing rate with 50% achieving lasting fistula closure (Table 4). This result was significant (*P* = 0.019).

**Table 4:** Healing rate for each individual attempt at fistula closure

Variable	Total	Success, *n* (%)		*P*-value
Method				
ELC^a^	40	12	(30.0)	0.019*
Local repair^b^	51	17	(33.3)	
Gracilis	10	5	(50.0)	
Others	21	1	(4.8)	
Total	122	35^c^	(28.7)	

^a^Excision and layered closure

^b^Local repairs included ERAF, LIFT and sphincteroplasty

^c^Two fistulas healed without having additional definitive procedures performed

Minor complications after index surgery occurred in 15 cases (27.3%). The majority were of Clavien-Dindo Grade 1 (*n* = 8, 14.5%), including postoperative pain that needed additional analgesics. Seven patients (12.7%) suffered Grade 2 complications. Six women were treated with antibiotics because of wound infection. One patient was treated with antibiotics for a lower urinary tract infection. One patient (1.8%) suffered a Grade 3b complication after a gracilis muscle transposition in whom bleeding from a superficial artery in the perineal wound required surgical intervention under general anesthesia.

### Seton and stoma

Index surgery was performed under protection of a stoma in 15 cases (27.3%). Ten of these were referred patients who received their protective stomas at the referring clinic. Another 12 patients received a stoma during the course of this study after index surgery, eight of which were performed at the referring clinic. Nine patients did not have a stoma closure during this study and four received a permanent stoma because of their RVF. Thirteen patients (23.6%) had a draining seton placed before their index surgery. A protective stoma had no influence on healing rate neither for index surgery (21.4% compared to 29.3%, *P* = 0.734) nor for final results (53.3% compared to 72.5%, *P* = 0.185). Patients drained with a seton before index surgery did not differ concerning healing rate after index surgery (15.4% compared to 31%. *P* = 0.477) or the final healing rate (76.9% compared to 64.3%, *P* = 0.510).

### Quality of life and patients’ assessments

**Fig. 3:**
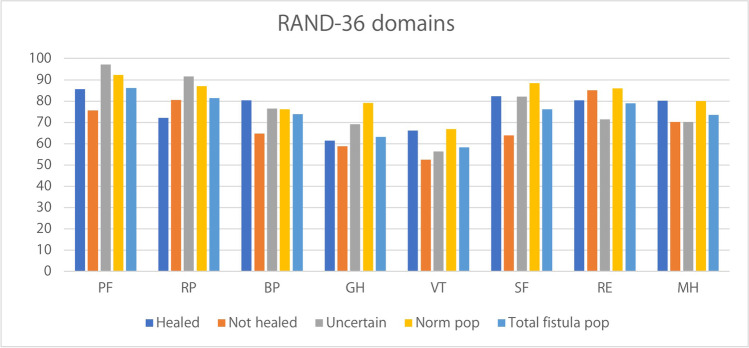
Mean RAND-36 domain scores (x-axis) for three categories of fistula patients (healed, unhealed, and uncertain healing, all matching the patient’s opinion) and the average score for the entire fistula population compared to the total Swedish population (Norm pop). PF = physical functioning; RP = role-physical; BP = bodily pain; GH = general health; VT = vitality; SF = social functioning; RE = role-emotional; MH = mental health

Of the 55 patients, 33 (17 healed, nine not healed, and seven uncertain) completed the questionnaires. Reasons for non-participation were the following: one patient deceased, 17 not giving consent, and another four were non-responders. In addition, two patients were excluded from the calculation of PCS and MCS due to incomplete questionnaires. Patients who stated that their fistula was unhealed reported numerically lower (non-significant *P*-value) than both healed patients and the normal population in 6/8 RAND-36 domains (Fig. 3). Our study population scored lower than the general Swedish population in all RAND-36 domains. There was no significant difference in the mean PCS or MCS when comparing patients with perceived healed and unhealed fistulation (70.6 SD25.0 vs 64.7 SD36.1, *P* = 0.85 and 74.2 ± 18.3 vs 66.1 ± 17.8, *P* = 0.28).

## Discussion

Remarkably, a novel finding in the present study was that inflammatory-related fistulas were associated with a less favorable prognosis when compared to the traumatic fistulas. Some previous studies suggest a poorer healing rate in infectious fistulas [1, 15], whereas others have shown opposite results [16]. However, none of the studies referred to have been able to show a significant difference between these etiologies. It could be suggested that the increased degree of inflammation possibly involved in anastomotic, infectious, and Crohn’s related fistulas causes the relatively lower healing rates when compared to fistulas of traumatic origin such as obstetric and iatrogenic RVF.

The healing rate for initial fistula closure was 25.5%, but repeated procedures led to a final healing rate of 67.3%. Even when final healing of the fistula is accomplished, quality of life might remain affected, as demonstrated by our study and earlier ones [1, 7].

The results of this study were roughly similar to previously published results.[5, 9, 17–22]. Results for index surgery at our department also concurred with those of other studies. With a majority of our patients being referred from other clinics, and many having undergone earlier attempts at fistula closure, it is possible that a great proportion of the fistulas could be considered complex. Differences in study populations regarding etiologies, fistula characteristics, and follow-up time make direct comparison between studies difficult. In a meta-analysis by Göttgens et al., healing rates ranged from 20 to 100% [9]. In the same study, the endorectal advancement flap procedure (ERAF) had an average closure rate of 60% [9]. Other local procedures include layered closure and LIFT treatment (ligation of inter-sphincteric fistula tract). A transvaginal approach with excision and layered closure obtained a closure rate of 67% [23]. Combining the LIFT procedure with a bioprosthetic mesh achieved a healing rate of 81% in a study of 27 patients [24]. The use of biomaterials, for example using a bioprosthetic mesh or a plug, has been examined in other studies. Closure rates have varied between 20 and 86% in smaller studies [9, 24, 25]. In complicated and recurrent RVF, abdominal approaches or a transposition of muscle such as gracilis transpositions can be used. In a systemic review, closure rates ranged from 43 to 100% using the gracilis transpositions [9].

Our study showed and average healing rate of 28.7% on each surgical attempt. The healing rate was slightly better for ELCs and local repairs. Gracilis transposition had a superior healing rate of 50%. One could suggest more liberal use of this method given these results but risks for side effects associated with more extensive surgery should also be considered. Many patients could still benefit from this practice earlier in the surgical strategy.

Limitations of this study include its retrospective nature and the heterogeneity of the cohort. The lack of preoperative RAND-36 scores is another shortcoming. Strengths of this study include a relatively large study population for a rather rare disorder, with outcome measures including both healing rate and HRQoL. Our results also illustrate the cumulative effect of repeated surgery for patients whose first attempt at RVF repair did not result in fistula healing.

Our study showed no significant difference for patients treated under a protective stoma. This is consistent with most previously published data [1, 2, 10, 11]. Stoma protection is still debated, as a stoma could theoretically support the healing process by protecting the repair from mechanical tear and inflammation. Stomas are often utilized with complicated fistulation but there is a lack of prospective studies. Moreover, a diverting stoma is often used to decrease the RVF symptoms.

In accordance with the literature, our study found that a major cause of RVF is obstetric injury [3, 26–30]. An overall decline in repairs of RVF was seen between 1979 and 2006 in the USA. Changes in obstetric care including increasing rates of cesarean delivery are thought to be a reason behind this trend [27]. The proportion of infection-related fistulas compared to fistulas of obstetric origin is higher in our study than in many earlier published studies. One possible reason is a difference in referral patterns, where some of the obstetric fistulas are handled by gynecologists and never reach our clinic. Another possible explanation is that our selection of patients is made on quite recent material and therefore reflects an ongoing shift towards declining occurrence of obstetric fistulas in high-income countries.

RVFs as a result of Crohn’s disease have historically been difficult to manage. With advances in medical therapy, treatment options for this subgroup have increased. Our results show that fistulas caused by Crohn’s disease still have a poor prognosis compared to obstetric fistulas. Other recently published studies have reached the same conclusion [5, 7, 31].

Although no significant difference in healing after index surgery was observed when analyzing surgical approach, at final follow-up, patients with an index procedure using a transperineal or transrectal approach had a better final healing rate. There are many possible explanations, one being that a patient with a surgical approach from the “others” group had more complicated fistulation at times treated with transvaginal or abdominal procedures more commonly used in higher fistulation. It is therefore possible that a larger proportion of this group had high fistulation which (shown in this study) predisposes to worse outcome. Further studies with larger study populations are needed to allow detailed comparison between the different methods allowing a subclassification with results for each method depending on fistula etiology and characteristics.

Most RVF repair is performed to close the fistula and thereby improve the physical well-being of the patient. In two studies performed by El-Gazzaz et al., no significant difference in quality of life or sexual function was shown between patients with healed and persistent or recurrent RVF [1, 7]. A small study of a Martius flap for low RVF revealed similar results [32]. A recent study by Picciariello et al. showed a significant improvement in all but two of the SF-36 domains [33]. However, the study examined the results of both RVF and recto-urethral fistulation and included both men and women. A study by Lefevre et al. showed significantly altered quality of life and sexual activity postoperatively irrespective of healing [34]. The extent of the positive impact upon HRQoL with successful surgery is left unanswered. None of the cited studies presented any example of fistula specific questionnaires which are, to our knowledge, not commonly used. More specific and standardized RVF-related questions might further aid in properly evaluating the surgical results.

Our results show that patients who perceived persistent fistulation score numerically lower in all but two RAND-36 domains compared to patients who consider their RVF healed. Furthermore, both groups scored numerically lower than the general population in every domain. The reason for persistently lower RAND-36 scores even in patients with healed fistulation might be remaining pelvic dysfunction after repeated surgery.

## Conclusion

Although the initial healing rate for RVF repair was low, two-thirds of the patients in our study achieved fistula healing with repeated surgery. Fistulas of traumatic origin (obstetric and iatrogenic) had a significantly better prognosis compared with those of inflammatory-related origin (anastomotic, infectious, and Crohn’s). The low healing rate after index surgery might be an argument for more extensive procedures early on in the surgical strategy. Our results as well as prior inconclusive results regarding the impact of fistula repair on patient well-being need to be evaluated in future studies, preferably with fistula symptom–specific questionnaires.
